# A valuable and affordable handheld ultrasound in combating COVID-19

**DOI:** 10.1186/s13054-020-03064-5

**Published:** 2020-06-12

**Authors:** Feng Qian, Xueqin Zhou, Jianqiao Zhou, Zhenhua Liu, Qian Nie

**Affiliations:** 1 Health Policy, Management, and Behavior, School of Public Health, University at Albany-State University of New York, Albany, USA; 2 Shanghai Soundwise Healthcare, Shanghai, China; 3 Shanghai Jiao Tong University Affiliated Ruijin Hospital, Shanghai, China; 4 Chengdu University of Traditional Chinese Medicine Affiliated Hospital, Chengdu, China

## Abstract

The handheld ultrasound demonstrates clinical and economic value in combating COVID-19 based on interviews with frontline ultrasound physician and cardiologist as well as a national expert in medical ultrasound.

The coronavirus pandemic has killed over 360,000 people worldwide. Millions of frontline healthcare workers are at a much higher risk of becoming infected when they test suspected cases and treat confirmed COVID-19 patients. As Mr. Bill Gates emphasizes the importance of “global innovation as the key” in combating COVID-19 [1], we hereby report a clinically valuable and economically affordable handheld ultrasound used in China. We conducted semi-structured interviews with two frontline physicians (Drs. Liu and Nie) and one national leading physician in medical ultrasound (Dr. Zhou) to capture their experience, usage, and views on the handheld ultrasound.

Dr. Liu was an ultrasound physician dispatched from Shanghai to Wuhan and had worked at a COVID-19 designated tertiary hospital during February–March 2020. He performed a total of 50 lung scan tests on hospitalized COVID-19 patients (24 mild (48%), 17 moderate (34%), and 9 severe (18%)) using the handheld ultrasound. Dr. Nie as a cardiologist had performed 10 heart scan tests with the handheld ultrasound on hospitalized patients at a COVID-19 designated tertiary hospital in Chengdu. Both doctors supported the use of the handheld ultrasound (see Fig. 1) because it greatly helped ensure physician safety in hospital settings and availed physicians to perform ultrasound scans after wearing the personal protection equipment.

**Fig. 1:**
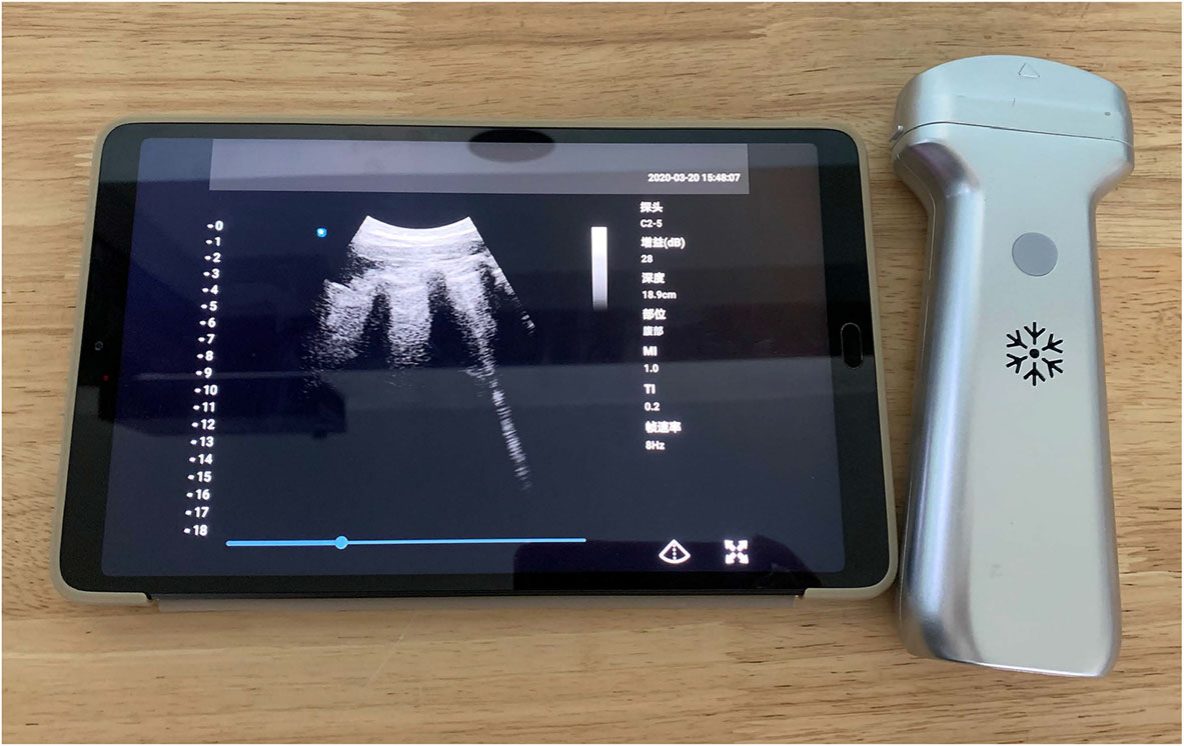
The handheld ultrasound device

Drs. Liu and Zhou commented that this handheld ultrasound generated comparable quality lung and heart images and boasted advantages of being small (probe, 17.9 cm × 7.5 cm × 3.4 cm), light (probe, 300 g), affordable (RMB 10,000–20,000 equivalent US$ 1412–2824), low test fee (RMB 70–100 equivalent US$ 10–15), artificial intelligence–assisted diagnosis, wireless feature, and battery rechargeable. So, it was easier for physicians to carry, operate, and disinfect. And it did not require much space in the hospital room and power sockets to be connected to the power cord to perform ultrasound scans. This helped reduce the risk of spreading the coronavirus as the power cord and cables would contact the surrounding environment which might be contaminated with coronavirus on the surface.

Dr. Liu said that it took less than 20 min for him to get used to using this device for performing a lung scan. After that, the average operation time for a mild or moderate case decreased to 5 min and for a severe case dropped to 10 min. Drs. Liu and Zhou mentioned that although the images’ quality was not as good as those from the CT lung scans, using this device avoided the need to move patients from the hospital room to imaging department, assisted bedside invasive procedures. Most importantly, it enabled physicians to detect the early signs of COVID-19 patient’s deterioration, for example, lower extremity deep vein thrombosis. Compared with chest X-ray, handheld ultrasound could provide more clinical information such as pulmonary artery pressure.

Interestingly, Dr. Liu said that the COVID-19 patients were very receptive to the handheld ultrasound as they thought it looked more sophisticated and stylish.

Dr. Nie emphasized the unique value of the handheld ultrasound in making differential diagnosis. It took him 2 min to complete a heart scan to rule out the possibility of his patient suffering from acute myocardial infarction or acute aortic dissection. Given that COVID-19 is associated with myocardial injury and other cardiovascular damages and that a substantial number of patients with heart attacks were found to delay emergency medical care due to their fear of COVID-19, an effective and efficient diagnostic test which can help physicians with diagnosis is extremely valuable [2, 3].

As advocated in recent literature [4, 5], the handheld ultrasound demonstrates clinical and economic value in combating COVID-19. Technological innovations such as this innovative device will help us defeat COVID-19 globally.

## Data Availability

Not applicable.
